# Analytical Solution to the Generalized Complex Duffing Equation

**DOI:** 10.1155/2022/2711466

**Published:** 2022-11-28

**Authors:** Alvaro H. Salas S, Gilder Cieza Altamirano, Lorenzo J. Martínez H

**Affiliations:** ^1^Universidad Nacional de Colombia, Fizmako Research Group, Bogotá, Colombia; ^2^Universidad Nacional Autónoma de Chota, Cajamarca, Peru; ^3^Fizmako Research Group, Bogotá, Colombia; ^4^Universidad de Caldas, Department of Mathematics and Statistics, Manizales, Colombia

## Abstract

Future scientific and technological evolution in many areas of applied mathematics and modern physics will necessarily depend on dealing with complex systems. Such systems are complex in both their composition and behavior, namely, dealing with complex dynamical systems using different types of Duffing equations, such as real Duffing equations and complex Duffing equations. In this paper, we derive an analytical solution to a complex Duffing equation. We extend the Krýlov–Bogoliúbov–Mitropólsky method for solving a coupled system of nonlinear oscillators and apply it to solve a generalized form of a complex Duffing equation.

## 1. Introduction

Numerous scholars have effectively used the theory of linear oscillations to analyze and model oscillatory devices. However, nonlinear behavior can be found in a wide range of real applications. Thus, scholars from several fields of science explore nonlinear systems and try to model and investigate these complicated systems in order to find solutions and explanations to some mysterious problems, whether in the manufacture of small and large machines or electronic chips. Consequently, nonlinear oscillation is one of the most popular and widely researched fields due to its diverse applications in automobiles, sensing, microscale and nanoscale, fluid and solid interaction, nonlinear oscillations in plasma physics, bioengineering, and nonlinear oscillations in optics. There are many different and various equations of motion that are used to model several nonlinear oscillations in different physical and engineering systems. The Duffing-type equation is one of the most famous and important equations that succeeded in explaining many different oscillations in different engineering, physical systems, and statistical mechanics.

The Duffing equation is a nonlinear second-order differential equation that describes an oscillator with complex, sometimes chaotic behavior. The Duffing equation was originally the result of Georg Duffing's systematic study of nonlinear oscillations. The behavior of the solution of the Duffing equation easily changes depending on the initial value and the polynomial coefficients, and it is difficult to predict its solution. To clarify the behavior of the solution, research based mainly on numerical analysis with high-precision calculations is conducted. Interest in the equation was later revived with the advent of chaos theory. Since then, the system has come to be regarded as one of the prototype systems in chaos theory, and related equations continue to find applications today, e.g., to describe the rolling of ships. The Duffing equation reads

(1) $$\begin{array}{c} \ddot{x}+\delta \dot{x}+\alpha x+\beta x^{3}=\gamma \;\cos\;\omega t\text{,} \end{array}$$

where *x*(*t*) is the displacement at time *t* and the term *γ* cos *ωt* represents a sinusoidal driving force. The cubic term describes an asymmetry in the restoring force of a spring that softens or stiffens as it is stretched. One of the most remarkable results of dynamical systems theory is the ubiquitousness of chaotic behavior in nonlinear systems. Deterministic chaos has been observed both in mathematical models and in real physical systems. Although, from the point of view of the applications, chaotic behavior can have positive effects, improving, for example, mixing processes in chemical reactions, in other situations, such behavior can have harmful consequences, as is the case in different fields of engineering: aerodynamics, electronic circuits, and magnetic confinement of plasmas.

Some recent works on complex chaos have focused on solving complex nonlinear differential equations, complex chaos control and synchronization, and so on. For example, Cveticanin developed an approximate analytic approach for solving strong nonlinear differential equations of the Duffing-type with a complex-valued function. Furthermore, excellent agreement is found between the analytic and numerical results.

In [1], authors considered the following complex Duffing equation for modelling complex signal detection:

(2) $$\begin{array}{c} \ddot{z}+k\dot{z}-z+\varepsilon z{\left|z\right|}^{2}=\gamma \;\exp\;\left(\sqrt{-1}t\right)\text{,} \end{array}$$

where $z=x+\sqrt{-1}y$ is a complex function, *k*, *ε*, and *γ* ≥ 0 are the real parameters, and the dots are the time derivatives. Its dynamical behavior was analyzed. Based on the proposed (2), they constructed a complex chaotic oscillator detection system to detect complex signals in noise. They investigated the influence of noise on the detection system and the detection performance of the system for complex signals.

In their work [2], the authors considered a complex Duffing system subjected to nonstationary random excitation of the form

(3) $$\begin{array}{c} \ddot{z}+2\omega \xi \dot{z}+\omega^{2}z+\epsilon z{\left|z\right|}^{2}=\alpha F\left(t\right)\text{,} \end{array}$$

where *z*=*z*(*t*) is a complex function, $\alpha =1+\sqrt{-1}$, *ω*, *ξ* represent the natural frequency and damping coefficient, respectively, *ϵ* is the small perturbation parameter and nonlinearity strength, and *F*(*t*) is a random function. This equation with *F*(*t*)=0 has connection to the complex nonlinear Schrodinger equation which appears in many important fields of physics. Authors in [2] investigated the mean square response of a complex Duffing system subjected to nonstationary random excitation using the Wiener–Hermite expansion method combining the perturbation technique.

In 2001, Mahmoud et al. [3] presented the following complex Duffing equation:

(4) $$\begin{array}{c} \ddot{z}-\alpha z+\varepsilon z{\left|z\right|}^{2}=\gamma \;\exp\;\left(\sqrt{-1}\omega t\right)\text{.} \end{array}$$

Based on the work in [3], Li et al. [4] studied the problem of chaos control for a complex Duffing oscillation system. In general, few works are devoted to the complex Duffing equation.

In this paper, we will consider the following complex Duffing equation:

(5) $$\begin{array}{c} \ddot{z}+2\varepsilon \dot{z}+\alpha z+\beta z{\left|z\right|}^{2}+\gamma {\bar{z}}^{3}=f_{1}\left(t\right)+\sqrt{-1}f_{2}\left(t\right),z=z\left(t\right)\text{.} \end{array}$$

To our best knowledge, no work has been devoted to seeking analytical solutions to the complex Duffing equation. This is precisely the main objective of the present paper.

## 2. Undamped and Unforced Complex Duffing Equation

Let us consider the i.v.p.

(6) $$\begin{array}{c} \ddot{z}+\alpha z+\beta z{\left|z\right|}^{2}+\gamma {\bar{z}}^{3}=0,z\left(0\right)=x_{0}+\sqrt{-1}y_{0}\;an\;d\;z^{\prime }\left(0\right)={\dot{x}}_{0}+\sqrt{-1}{\dot{y}}_{0}\text{.} \end{array}$$

Let

(7) $$\begin{array}{c} z\left(t\right)=x\left(t\right)+\sqrt{-1}y\left(t\right)\text{.} \end{array}$$

Then,

(8) $$\begin{array}{c} x^{\prime \prime }\left(t\right)+x\left(t\right)\left(\alpha +\left(\beta -3\gamma \right)y{\left(t\right)}^{2}\right)+\left(\beta +\gamma \right)x{\left(t\right)}^{3}=0\text{,}\\ y^{\prime \prime }\left(t\right)+y\left(t\right)\left(\alpha +\left(\beta -3\gamma \right)x{\left(t\right)}^{2}\right)+\left(\beta +\gamma \right)y{\left(t\right)}^{3}=0\text{.} \end{array}$$

Assume that *x*=*x*(*t*) and *y*=*y*(*t*) obey some Duffing equations:

(9) $$\begin{array}{c} \ddot{x}+px+qx^{3}=0,\ddot{y}{+ry+sy}^{3}=0\text{.} \end{array}$$

Then,

(10) $$\begin{array}{c} x\left(t\right)\left(\alpha -p+\left(\beta -3\gamma \right)y{\left(t\right)}^{2}\right)+x{\left(t\right)}^{3}\left(\beta +\gamma -q\right)=0\text{,}\\ \left(\alpha -r\right)y\left(t\right)+y{\left(t\right)}^{3}\left(\beta +\gamma -s\right)+\left(\beta -3\gamma \right)x{\left(t\right)}^{2}y\left(t\right)=0\text{.} \end{array}$$

Equating to zero, the coefficients of *x*(*t*) and *y*(*t*) in (10) give

(11) $$\begin{array}{c} p=\alpha ,q=\frac{4\beta }{3},r=\alpha ,s=\frac{4\beta }{3}an\;d\;\gamma =\frac{\beta }{3}\text{.} \end{array}$$

Thus,

(12) $$\begin{array}{c} \ddot{x}+\alpha x+\frac{4\beta }{3}x^{3}=0,x\left(0\right)=x_{0},x^{\prime }\left(0\right)={\dot{x}}_{0}\text{,}\\ \ddot{y}+\alpha y+\frac{4\beta }{3}y^{3}=0,y\left(0\right)=y_{0},y^{\prime }\left(0\right)={\dot{y}}_{0}\text{.} \end{array}$$

On the other hand, the exact solution to the i.v.p.

(13) $$\begin{array}{c} \ddot{u}+Au+Bu^{3}=0,u\left(0\right)=u_{0}\;an\;d\;u^{\prime }\left(0\right)={\dot{u}}_{0} \end{array}$$

is expressed as

(14) $$\begin{array}{c} u\left(t\right)=u_{0}cn\left(\sqrt{\omega }t,m\right)+\frac{{\dot{u}}_{0}}{\sqrt{\omega }}sn\left(\sqrt{\omega }t,m\right)dn\left(\sqrt{\omega }t,m\right)/1+b\;{sn}^{2}\left(\sqrt{\omega }t,m\right)\text{,} \end{array}$$

where

(15) $$\begin{array}{c} b=\frac{B\left(1-2m\right)u_{0}^{2}}{2A}-m\text{,}\\ \omega =\frac{A}{1-2m}\text{,}\\ m=\frac{1}{2}\left(1\pm \frac{A}{\sqrt{{\left(A+Bu_{0}^{2}\right)}^{2}+2B{\dot{u}}_{0}^{2}}}\right)\text{.} \end{array}$$

## 3. Solution to the General Complex Duffing Equation by Means of the Krýlov–Bogoliúbov–Mitropólsky Method

Let us consider the i.v.p.

(16) $$\begin{array}{c} \ddot{z}+2\varepsilon \dot{z}+\alpha z+\beta z{\left|z\right|}^{2}+\gamma {\bar{z}}^{3}=f_{1}\left(t\right)+\sqrt{-1}f_{2}\left(t\right),z\left(0\right)=z_{0\;}and\;z^{\prime }\left(0\right)={\dot{z}}_{0}\text{.} \end{array}$$

Here, *α*, *β*, and *γ* are the real numbers. *f*_1_(*t*) and *f*_2_(*t*) are the real-valued functions, and $z\left(t\right)=x\left(t\right)+\sqrt{-1}y\left(t\right)$. The system (16) may be written in the form

(17) $$\begin{array}{c} \left\{\begin{array}{c} \ddot{x}+\alpha x+2\varepsilon \dot{x}+\left(\beta -3\gamma \right)xy^{2}+\left(\beta +\gamma \right)x^{3}-f_{1}\left(t\right)=0\text{,}\\ \ddot{y}+\alpha y+2\varepsilon \dot{y}+\left(\beta -3\gamma \right)x^{2}y+\left(\beta +\gamma \right)y^{3}-f_{2}\left(t\right)=0\text{.} \end{array}\right. \end{array}$$

The initial conditions are

(18) $$\begin{array}{c} x\left(0\right)=x_{0},y\left(0\right)=y_{0},x^{\prime }\left(0\right)={\dot{x}}_{0},y^{\prime }\left(0\right)={\dot{y}}_{0}\text{.} \end{array}$$

Let us consider the following *p*-problem:

(19) $$\begin{array}{c} \left\{\begin{array}{c} \ddot{x}+\alpha x+p\left[2\varepsilon \dot{x}+\left(\beta -3\gamma \right)xy^{2}+\left(\beta +\gamma \right)x^{3}-f_{1}\left(t\right)\right]=0\text{,}\\ \ddot{y}+\alpha y+p\left[2\varepsilon \dot{y}+\left(\beta -3\gamma \right)x^{2}y+\left(\beta +\gamma \right)y^{3}-f_{2}\left(t\right)\right]=0\text{.} \end{array}\right. \end{array}$$

The solution is assumed to be in the ansatz form

(20) $$\begin{array}{c} x\left(t\right)=a\left(t\right)\cos\;\left(\psi \left(t\right)\right)+\overset{N}{\underset{n=1}{\sum }}p^{n}v_{n}\left(a\left(t\right),b\left(t\right),\psi \left(t\right),\Psi \left(t\right)\right)\text{,}\\ y\left(t\right)=b\left(t\right)\cos\;\left(\Psi \left(t\right)\right)+\overset{N}{\underset{n=1}{\sum }}p^{n}w_{n}\left(a\left(t\right),b\left(t\right),\psi \left(t\right),\Psi \left(t\right)\right)\text{,}\\ a^{\prime }\left(t\right)=\overset{N}{\underset{n=1}{\sum }}p^{n}A_{n}\left(a\left(t\right)\right)\text{,}\\ \psi^{\prime }\left(t\right)=\sqrt{\alpha }+\overset{N}{\underset{n=1}{\sum }}p^{n}\varphi_{n}\left(a\left(t\right)\right)\text{,}\\ b^{\prime }\left(t\right)=\overset{N}{\underset{n=1}{\sum }}p^{n}B_{n}\left(b\left(t\right)\right)\text{,}\\ \Psi^{\prime }\left(t\right)=\sqrt{\alpha }+\overset{N}{\underset{n=1}{\sum }}p^{n}\phi_{n}\left(b\left(t\right)\right)\text{.} \end{array}$$

We choose the solutions in order to avoid the presence of the so-called secularity terms. Solving the odes gives

(21) $$\begin{array}{c} \varphi_{1}\left(a\right)=\frac{3\left(\beta +\gamma \right)+2b^{2}\left(\beta -3\gamma \right)}{8\sqrt{\alpha }}a^{2},\phi_{1}\left(a\right)=\frac{\left(5\beta -3\gamma \right)}{8\sqrt{\alpha }}a^{2}\text{,}\\ A_{1}\left(a\right)=-a\varepsilon ,B_{1}\left(b\right)=-b\varepsilon \text{,}\\ v_{1}\left(a,\psi ,\Psi \right)=\frac{1}{32\alpha }\left(a^{3}\left(\beta +\gamma \right)\cos\;\left(3\psi \right)-4ab^{2}\left(\beta -3\gamma \right)\cos\;\left(2\Psi \right)\left(2\psi \;\sin\left(\psi \right)+\cos\left(\psi \right)\right)+32f_{1}\left(t\right)\right)\text{,}\\ w_{1}\left(b,\psi ,\Psi \right)=\frac{1}{32\alpha }\left(-4a^{2}b\left(\beta -3\gamma \right)\cos\;\left(2\psi \right)\left(2\Psi \;\sin\left(\Psi \right)+\cos\left(\Psi \right)\right)+b^{3}\left(\beta +\gamma \right)\cos\;\left(3\Psi \right)+32f_{2}\left(t\right)\right)\text{,}\\ \dot{a}=-a\varepsilon p,\dot{b}=-b\varepsilon p\text{,}\\ \dot{\psi }=\sqrt{\alpha }+\frac{p}{8\sqrt{\alpha }}\left(3a^{2}\beta +3a^{2}\gamma +2b^{2}\beta -6b^{2}\gamma \right),\dot{\Psi }=\sqrt{\alpha }+\frac{p}{8\sqrt{\alpha }}\left(2a^{2}\beta -6a^{2}\gamma +3b^{2}\beta +3b^{2}\gamma \right)\text{.} \end{array}$$

The approximate analytical solution is obtained by letting *p*=1. It reads

(22) $$\begin{array}{c} x\left(t\right)=a\;\cos\left(\psi \right)+\frac{1}{32\alpha }\left(a^{3}\left(\beta +\gamma \right)\cos\;\left(3\psi \right)-4ab^{2}\left(\beta -3\gamma \right)\cos\;\left(2\Psi \right)\left(2\psi \;\sin\left(\psi \right)+\cos\left(\psi \right)\right)+32f_{1}\left(t\right)\right)\text{,}\\ y\left(t\right)=b\;\cos\left(\Psi \right)+\frac{1}{32\alpha }\left(-4a^{2}b\left(\beta -3\gamma \right)\cos\;\left(2\psi \right)\left(2\Psi \;\sin\left(\Psi \right)+\cos\left(\Psi \right)\right)+b^{3}\left(\beta +\gamma \right)\cos\;\left(3\Psi \right)+32f_{2}\left(t\right)\right)\text{.} \end{array}$$

The expressions for*a*, *b*, *ψ*, an d Ψ are

(23) $$\begin{array}{c} a=a\left(t\right)=c_{0}\exp\;\left(-\varepsilon t\right),b=b\left(t\right)=d_{0}\exp\;\left(-\varepsilon t\right)\text{,}\\ \psi \left(t\right)=\frac{1}{8\sqrt{\alpha }}\left(\frac{\exp\;\left(-\varepsilon t\right)\sinh\;\left(\varepsilon t\right)\left(3c_{0}^{2}\left(\beta +\gamma \right)+2d_{0}^{2}\left(\beta -3\gamma \right)\right)}{\varepsilon }+8\alpha t\right)+c_{1}\text{,}\\ \Psi \left(t\right)=\frac{1}{8\sqrt{\alpha }}\left(\frac{\exp\;\left(-\varepsilon t\right)\sinh\;\left(\varepsilon t\right)\left(2c_{0}^{2}\left(\beta -3\gamma \right)+3d_{0}^{2}\left(\beta +\gamma \right)\right)}{\varepsilon }+8\alpha t\right)+d_{1}\text{.} \end{array}$$

The constants *c*_0_, *c*_1_, *d*_0_, and *d*_1_ are obtained from the initial conditions.

The obtained solution is valid for *α* > 0. Let *α* < 0 for the sake of simplicity; we will consider only the case when *γ*=0. Let us change *α* to −*α*. We are given that

(24) $$\begin{array}{c} \ddot{z}+2\varepsilon \dot{z}-\alpha z+\beta z{\left|z\right|}^{2}=f_{1}\left(t\right)+\sqrt{-1}f_{2}\left(t\right),z\left(0\right)=z_{0}an\;d\;z^{\prime }\left(0\right)={\dot{z}}_{0}\text{.} \end{array}$$

In the case when *ε*=0 and *f*_1_(*t*)=*f*_2_(*t*) ≡ 0, direct calculations show that the following function will be the exact solution to $\ddot{z}-\alpha z+\varepsilon z{\left|z\right|}^{2}=0$:

(25) $$\begin{array}{c} z\left(t\right)=c_{0}dn\left(\sqrt{\frac{\varepsilon \left(c_{0}^{2}+d_{0}^{2}\right)}{2}}t+c_{1}|\frac{2\left(\alpha -\varepsilon c_{0}^{2}-\varepsilon d_{0}^{2}\right)}{\varepsilon \left(c_{0}^{2}+d_{0}^{2}\right)}\right)\\ +\sqrt{-1}d_{0}dn\left(\sqrt{\frac{\varepsilon \left(c_{0}^{2}+d_{0}^{2}\right)}{2}}t+d_{1}|\frac{2\left(\alpha -\varepsilon c_{0}^{2}-\varepsilon d_{0}^{2}\right)}{\varepsilon \left(c_{0}^{2}+d_{0}^{2}\right)}\right)\text{.} \end{array}$$

The constants *c*_0_, *c*_1_, *d*_0_, and *d*_1_ are determined from the initial conditions:

(26) $$\begin{array}{c} z\left(0\right)=x_{0}+\sqrt{-1}y_{0}\;an\;d\;z^{\prime }\left(0\right)={\dot{x}}_{0}+\sqrt{-1}{\dot{y}}_{0}\text{.} \end{array}$$

Let us solve the general case. Assume the solution in the ansatz form:

(27) $$\begin{array}{c} z\left(t\right)=r+x\left(t\right)+\sqrt{-1}y\left(t\right),r^{2}=\frac{\alpha }{\beta }\text{.} \end{array}$$

Then,

(28) $$\begin{array}{c} \ddot{x}+2\alpha x+2\varepsilon \dot{x}+3\beta rx^{2}+\beta ry^{2}+\beta x^{3}+\beta xy^{2}=f_{1}\left(t\right)\text{,}\\ \ddot{y}+2\varepsilon \dot{y}+2\beta rxy+\beta x^{2}y+\beta y^{3}=f_{2}\left(t\right)\text{.} \end{array}$$

We may solve the above system using the KBM method. To this end, we consider the following *p*-problem:

(29) $$\begin{array}{c} \ddot{x}+2\alpha x+p\left[2\varepsilon \dot{x}+3\beta rx^{2}+\beta ry^{2}+\beta x^{3}+\beta xy^{2}-f_{1}\left(t\right)\right]=0\text{,}\\ \ddot{y}+y+p\left[-y+2\varepsilon \dot{y}+2\beta rxy+\beta x^{2}y+\beta y^{3}-f_{2}\left(t\right)\right]=0\text{.} \end{array}$$

Proceeding in the same way as we did in the first part, we obtain the following first-order approximation:

(30) $$\begin{array}{c} \begin{array}{c} x\left(t\right)=\frac{e^{-3\varepsilon t}}{64\alpha }\left(\begin{array}{c} \beta c_{0}^{3}\cos\;\left(3\psi \right)-4\beta c_{0}d_{0}^{2}\cos\;\left(2\phi \right)\left(2\psi \;\sin\left(\psi \right)+\cos\left(\psi \right)\right)\\ +16\beta re^{\varepsilon t}\left(c_{0}^{2}\left(\cos\;\left(2\psi \right)-3\right)-2d_{0}^{2}\cos^{2}\left(\phi \right)\right)+64\alpha c_{0}e^{2\varepsilon t}\;\cos\left(\psi \right) \end{array}\right)+\frac{1}{2\alpha }f_{1}\left(t\right),\\ y\left(t\right)=\frac{1}{32}d_{0}e^{-3\varepsilon t}\left(\begin{array}{c} -8\beta c_{0}\left(\phi \;\sin\left(\phi \right)+\cos\left(\phi \right)\right)\left(c_{0}\cos\;\left(2\psi \right)+4re^{\varepsilon t}\;\cos\left(\psi \right)\right)\\ +\beta d_{0}^{2}\cos\;\left(3\phi \right)+32e^{2\varepsilon t}\;\cos\left(\phi \right) \end{array}\right)+f_{2}\left(t\right). \end{array} \end{array}$$

Here,

(31) $$\begin{array}{c} \psi =\psi \left(t\right)=\frac{e^{-2\varepsilon t}}{32\sqrt{\alpha }\varepsilon }\left(4\varepsilon \left(8\sqrt{\alpha }c_{1}e^{2\varepsilon t}+\sqrt{2}t\left(\beta d_{0}^{2}+8\alpha e^{2\varepsilon t}\right)\right)+3\sqrt{2}\beta c_{0}^{2}\left(e^{2\varepsilon t}-1\right)\right)\text{,}\\ \phi =\phi \left(t\right)=\frac{1}{16}\left(4\beta c_{0}^{2}te^{-2\varepsilon t}+\frac{\beta d_{1}^{2}\left(3-3e^{-2\varepsilon t}\right)}{\varepsilon }+16d_{1}+8t\right)\text{.} \end{array}$$

The constants *c*_0_, *c*_1_, *d*_0_, and *d*_1_ are determined from the initial conditions:

(32) $$\begin{array}{c} z\left(0\right)=x_{0}+\sqrt{-1}y_{0}\;an\;d\;z^{\prime }\left(0\right)={\dot{x}}_{0}+\sqrt{-1}{\dot{y}}_{0}\text{.} \end{array}$$

## 4. Applications

Let us check the accuracy of the obtained results in concrete examples.

**Example 1 .**

Let

(33) $$\begin{array}{c} z^{\prime \prime }\left(t\right)+3z\left(t\right)+0.04z^{\prime }\left(t\right)+z\left(t\right){\left|z\left(t\right)\right|}^{2}+0.2{\left(z{\left(t\right)}^{*}\right)}^{3}=F\left(t\right)\text{,}\\ F\left(t\right)≔=0.1cn\left(0.1t|0.9\right)+\left(0.+0.1i\right)sn\left(0.1t|0.9\right)\text{.}\\ z\left(0\right)=0\wedge z^{\prime }\left(0\right)=0\text{.} \end{array}$$

See Figures 1–3.

**Example 2 .**

Let

(34) $$\begin{array}{c} \overset{.}{z}+2z+0.2\dot{z}+z{\left|z\right|}^{2}+0.2{\bar{z}}^{3}=0.1\;\cos\;\left(0.2t\right)-i\;\cos\;\left(0.1t\right)\wedge z\left(0\right)=0\wedge z^{\prime }\left(0\right)=0\text{.} \end{array}$$

The approximate analytical solution reads

(35) $$\begin{array}{c} z_{approx}\left(t\right)=x\left(t\right)+iy\left(t\right)\text{,} \end{array}$$

where

(36) $$\begin{array}{c} x=\left.e^{-0.3t}\left(\cos\right.\;\left(2\right.\left(\Psi \right)\left(6.23\right)E-6\psi \;\sin\;\left(\psi \right)+3.15E-6\;\cos\;\left(\psi \right)\right)\\ \left.-2.4E-6\;\cos\;\left(3\right)\left(\psi \right)-0.0501254e^{-0.1t}\;\cos\;\left(\psi \right)+0.05\;\cos\;\left(0.2\right)t\right)\\ y=\left.e^{-0.3t}\left(\;\right)\cos\;\left(2\right)\left(\psi \right)\left(-\right)6.3E-6\Psi \;\sin\;\left(\Psi \right)-3.15E-6\;\cos\;\left(\Psi \right)\right)\\ \left.+2.-4E-6\;\cos\;\left(3\right)\left(\Psi \right)+0.0501254e^{-0.1t}\;\cos\;\left(\Psi \right)-0.05\;\cos\;\left(0.1\right)t\right)\\ \psi =1.41421t-0.00488578e^{-0.2t}-0.0656304\text{.}\\ \Psi =1.41421t-0.00488578e^{-0.2t}-0.0656304\text{.} \end{array}$$

See Figures 4–6.

## 5. Conclusions

The nonlinear complex Duffing oscillators and many related oscillators, including the unforced undamped complex Duffing oscillator (CDO), the unforced damped CDO, and the forced damped CDO, have been analyzed using the ansatz method in order to find some approximations. For the unforced undamped CDO, the exact solution of the standard Duffing oscillator (DO) with the ansatz method was used for deriving an analytical approximation in terms of the Jacobi elliptic function. Also, the unforced damped CDO has been analyzed using the ansatz method, and with the help of the approximation of the unforced damped DO, an approximation in the form of a trigonometric form was obtained. Moreover, the forced damped CDO has been examined via the Krýlov–Bogoliúbov–Mitropólsky method (KBM), and a new analytical approximation in the form of a trigonometric formula has been derived. We demonstrated the way we may use the KBM in order to solve coupled systems of nonlinear oscillators. Other works related to nonlinear oscillators may be found in [5–13].

## Figures and Tables

**Figure 1:**
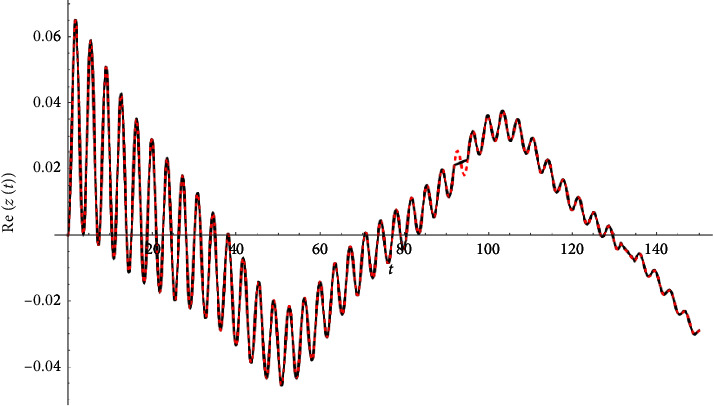
Real part compared with the Runge–Kutta numerical solution.

**Figure 2:**
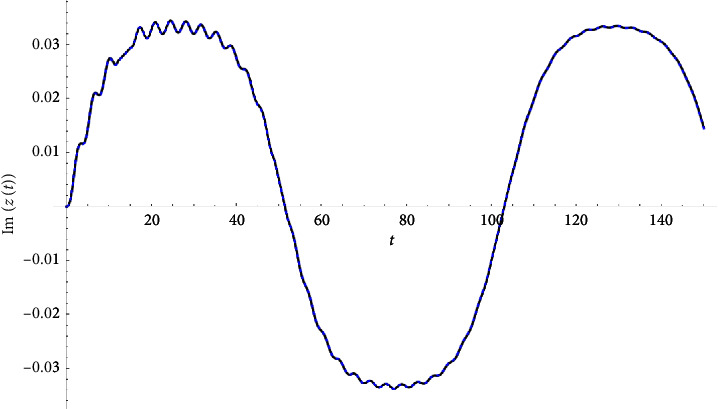
Imaginary part compared with the Runge–Kutta numerical solution.

**Figure 3:**
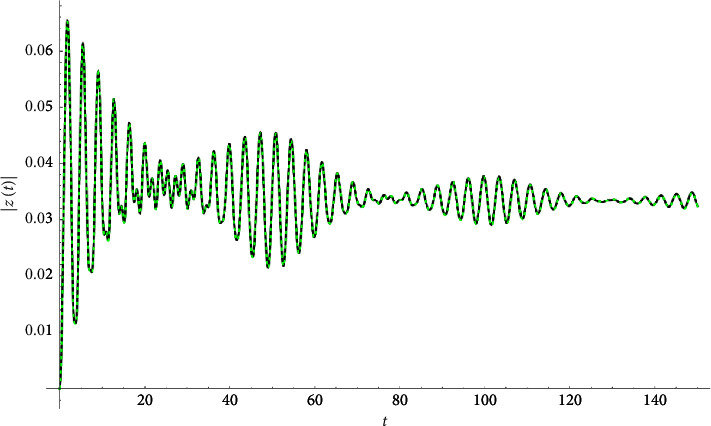
Absolute value compared with the Runge–Kutta numerical solution.

**Figure 4:**
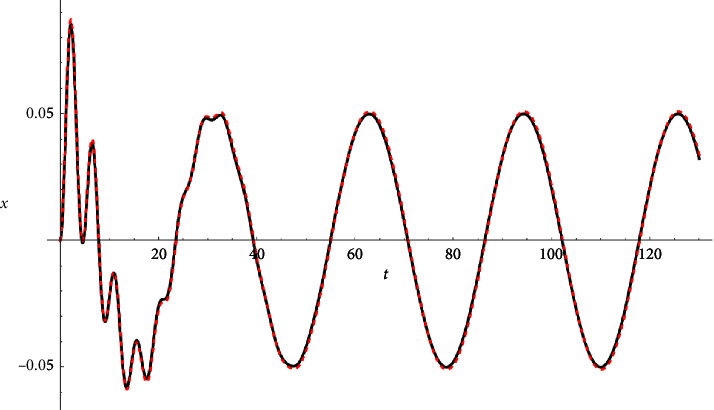
Real part compared with the Runge–Kutta numerical solution.

**Figure 5:**
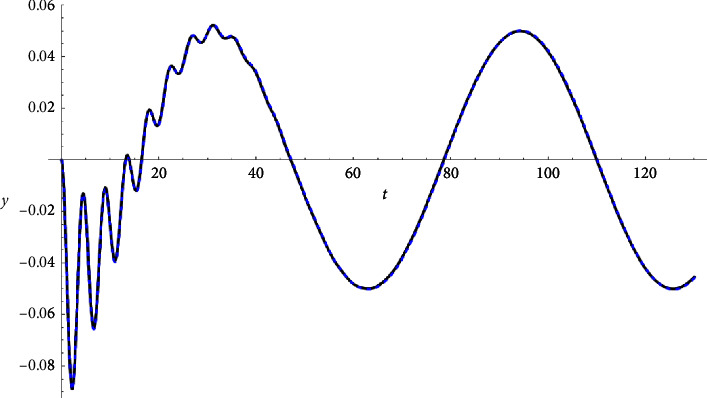
Imaginary part compared with the Runge–Kutta numerical solution.

**Figure 6:**
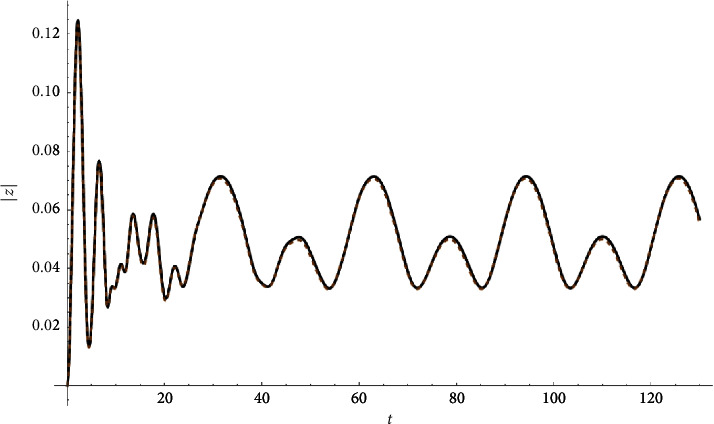
Absolute value |*z*| compared with the Runge–Kutta numerical solution.

## Data Availability

No data were used to support this study.
